# Shenqi fuzheng, an injection concocted from chinese medicinal herbs, combined with platinum-based chemotherapy for advanced non-small cell lung cancer: a systematic review

**DOI:** 10.1186/1756-9966-29-137

**Published:** 2010-10-22

**Authors:** Ju Dong, Shi-Yue Su, Min-Yan Wang, Zhen Zhan

**Affiliations:** 1 Institute of Basic Medical Science, Nanjing University of Traditional Chinese Medicine, Nanjing, P.R China; 2 Chuzhou City People's Hospital The Second Party, Chuzhou, P.R China; 3 Institute of Basic Medical Science, Nanjing University of Traditional Chinese Medicine, Nanjing, P.R China; 4 Institute of Basic Medical Science, Nanjing University of Traditional Chinese Medicine, Nanjing, P.R China

## Abstract

**Background:**

Platinum-based chemotherapy has been a standard therapy for advanced non-small cell lung cancer (NSCLC), but it has high toxicity. In China, Shenqi Fuzheng, a newly developed injection concocted from Chinese medicinal herbs has been reported that may increase efficacy and reduce toxicity when combined with platinum-based chemotherapy, but little is known about it outside of China. The aim of this study was to systematically review the existing clinical evidence on Shenqi Fuzheng Injection(SFI) combined with platinum-based chemotherapy for advanced NSCLC.

**Methods:**

Pubmed, Cochrane Library, EMBASE, CNKI, and CBM search were organized for all documents published, in English and Chinese, until April 2010. The randomized controlled clinical trials were selected based on specific criteria, in which a SFI plus platinum-based chemotherapy treatment group was compared with a platinum-based chemotherapy control group for patients with advanced NSCLC. The quality of studies was assessed by modified Jadad's scale, and Revman 4.2 software was used for data syntheses and analyses.

**Results:**

Twenty nine studies were included in this review based on our selection criteria. Of them, ten studies were of high quality and the rest were of low quality, according to the modified Jadad scale. The meta-analysis showed there was a statistically significant higher tumor response (RR, 1.19; 95% CI, 1.07 to 1.32; P = 0.001) and performance status ((RR, 1.57; 95% CI, 1.45 to 1.70; P < 0.00001); but lower severe toxicity for WBC (RR, 0.37; 95% CI, 0.29 to 0.47; P < 0.00001), PLT (RR, 0.33; 95% CI, 0.21 to 0.52; P < 0.00001), HB (RR, 0.44; 95% CI, 0.30 to 0.66; P < 0.0001) and nausea and vomiting (RR, 0.32; 95% CI, 0.22 to 0.47; P < 0.00001), when the SFI plus platinum-based chemotherapy treatment group was compared with the platinum-based chemotherapy control group. Sensitivity analysis was restricted to studies with the high quality, and the result was similar when the studies with low quality were excluded. Asymmetry was observed in a funnel plot analysis, and Egger's test also indicated an evidence of publication bias (*P *= 0.016).

**Conclusions:**

SFI intervention appears to be useful to increase efficacy and reduce toxicity when combined with platinum-based chemotherapy for advanced NSCLC, although this result needs to be further verified by more high-quality trials.

## Background

Lung cancer is the leading cause of cancer-related mortality around the world, of which non-small cell lung cancer (NSCLC) accounts for approximately 85% [1]. Moreover, most NSCLC cases already reach stages III and IV at the time of diagnosis indicating an advanced and often inoperable stage of NSCLC. Platinum-based chemotherapy has been a standard therapy and is widely accepted for treatment of advanced NSCLC [1,2]. The superiority of platinum-based chemotherapy over non-platinum-based chemotherapy has been proved by many randomized clinical trials. However, the resulting hematal and gastrointestinal toxicity, such as leukopenia, thrombopenia, nausea, vomiting and so on, have also been reported [3,4], which may seriously affect the patient's survival quality and curative effects. So, questions remain on how to best reduce the toxicity and enhance the curative effect of platinum-based chemotherapy.

In China, to reduce the toxicity and enhance the curative effect of platinum-based chemotherapy, many traditional Chinese medicinal herbs have been widely used combined with platinum-based chemotherapy for the treatment of advanced NSCLC, and some researchers[5,6] have found that combining Chinese medicinal herbs with platinum-based chemotherapy for the treatment of advanced NSCLC may improve survival, tumor response, and performance status, as well as reduce chemotherapy toxicity.

Shenqi Fuzheng is a newly developed injection concocted from two kinds of Chinese medicinal herbs: Radix Astragali (root of astragalus; Chinese name: huangqi) and Radix Codonopsis (root of Codonopsis pilosula; Chinese name: dangshen)[7,8], approved by the State Food and Drug Administration of the People's Republic of China in 1999 primarily as an antitumor injection to be manufactured and marketed in China [9,10]. Currently, there are many published trials about Shenqi Fuzheng Injection(SFI) combined with platinum-based chemotherapy for treatment of advanced NSCLC, some of which have shown that SFI may play an important role in the treatment of advanced NSCLC, could improve tumor response, performance status and reduce the toxicity of standard platinum-based chemotherapy. However, little is known about it outside of China, and there has not been a systematic evaluation until now. This paper presents a systematic review in an effort to clarify whether SFI in combination with platinum-based chemotherapy for advanced NSCLC really increases the efficacy and decreases the toxicity.

## Methods

### Search strategy

According to guidelines from the Cochrane collaboration [11], PubMed (1966 to April 2010); Cochrane Library (1988 to April 2010); EMBASE (1974 to April 2010); and Cochrane Central Register of Controlled Trials (1966 to April 2010); CBM (1978 to April 2010); CNKI(1984 to April 2010) were organized for search, and the following keywords were used: non-small-cell lung cancer, platinum-based chemotherapy, Shenqi Fuzheng injection, randomized controlled trials and multiple synonyms for each term. The publication languages were restricted to Chinese and English.

### Studies selection

Trials were included if they were randomized controlled trials comparing a SFI plus platinum-based chemotherapy treatment group with a platinum-based chemotherapy control group for patients with advanced NSCLC. Moreover, the reported data must have at least one of following outcomes: objective tumor response (the 4-point WHO scale [12] was adopted), performance status (the Karnofsky performance scale [13] was used and performance status was divided into 3 grades using a 10-point change as the cutoff), and toxicity (the 5-point WHO scale [12] was used), and the reported data also needed to have sufficient detail to permit the calculation of the risk ratios and it's 95% CIs for each outcome. Data expressed as medians were not included in this meta-analysis, and the duplicates, case series, and case reports were also excluded.

### Data extraction

All data on patient characteristics, treatment details, and clinical outcomes were independently abstracted and duplicated by two investigators (Ju Dong, Shi-Yue Su) using a standardized data collection form. Disagreements on study inclusion or data extraction were resolved by consensus of all coauthors. The outcome measures extracted were: objective tumor response, improved or stabilized performance status, and severe chemotherapy toxicity.

### Statistical analysis

Meta-analysis was done with Review Manager 4.2 (The Cochrane Collaboration, Oxford, UK) [11]. Relative ratio (RR) and 95% confidence intervals (CI) were calculated, hypothesis of homogeneity was not rejected, the fixed-effects model was used to calculate the summary relative ratio (RR), and the 95% CI. Otherwise, a random-effects model was used [14]. In this meta-analysis, three kind of following outcomes were calculated and analyzed appropriately.

#### 1. Objective tumor response

The rate of tumor response was calculated as the number of patients experiencing complete response and partial response divided by the total number of patients (complete response plus partial response plus no change plus progressive disease) in each group, The RR of tumor response was calculated as the rate of tumor response in the SFI combined with platinum-based chemotherapy treatment group divided by that in the platinum-based chemotherapy control group. Thus, a RR of more than 1 favors the SFI combined with platinum-based chemotherapy treatment group. This method has been recommended by Sutton et al [15].

#### 2. Improved or stable performance status

This is similar to the approach of Michael et al [5]. The rate of improved or stable performance status was calculated as the proportion of improved or stable performance status (>10-point increase plus no change) divided by the total (>10-point increase, plus no change, plus >10-point decrease). The RR of improved or stable performance status was analyzed as the rate of improved or stable performance status in the SFI combined with platinum-based chemotherapy treatment group, divided by this proportion in the platinum-based chemotherapy control group. Thus, a RR of more than 1 favors the SFI combined with platinum-based chemotherapy treatment group.

#### 3. Severe chemotherapy toxicity

Using the approach of Delbaldo et al [16], the rate of severe chemotherapy toxicity was defined as the number of patients experiencing severe toxicity (WHO grades 3 and 4) divided by the total number of patients (WHO grades 0, 1, 2, 3 and 4) in each group. The RR of severe chemotherapy toxicity was analyzed as the proportion of severe toxicity in the SFI combined with platinum-based chemotherapy treatment group divided by this proportion in the platinum-based chemotherapy control group. Thus, a RR of less than 1 favors the SFI combined with platinum-based chemotherapy treatment group.

### Study quality evaluation

Two reviewers (Ju Dong, Shi-Yue Su) independently graded each RCT/CCT using the modified Jadad scale[17]. The modified Jadad scale is an eight-item scale designed to assess randomization, blinding, withdrawals/dropouts, inclusion/exclusion criteria, adverse effects, and statistical analysis (table 1). The score for each article can range from 0 (lowest quality) to 8 (highest quality). Scores of 4-8 represent good to excellent (high quality) and 0 to 3 poor or low quality.

**Table 1:** The modified Jadad scale

Eight-item of the modified Jadad scale		Score
Was the study described as randomized?	Yes	+1
	No	0
Was the method of randomization appropriate?	Yes	+1
	No	-1
	Not described	0
Was the study described as blinding?^a^	Yes	+1
	No	0
Was the method of blinding appropriate?	Yes	+1
	No	-1
	Not described	0
Was there a description of withdrawals and dropouts?	Yes	+1
	No	0
Was there a clear description of the inclusion/exclusion criteria?	Yes	+1
	No	0
Was the method used to assess adverse effects described?	Yes	+1
	No	0
Was the methods of statistical analysis described?	Yes	+1
	No	0

a: double-blind got 1 score, single-blind got 0.5 score.

### Sensitivity analysis

Sensitivity analysis was used to assess how robust the results are to uncertain decisions or assumption about the data and the methods that were used [18]. To analyze the sensitivity of our study, some studies were excluded because they were of low quality (had a quality score of 3 or under 3) and thus may weaken the conclusions.

### Publication bias analysis

For the purposes of assessing the publication bias of this study, a funnel plot based on studies with data on objective tumor response (as this was the outcome with most studies included in meta-analysis) was graphed and Egger's test[19] was also performed.

## Results

### Study characteristics and quality

Twenty nine studies [20-48] were included in this review based on our selection criteria, encompassing 2,062 patients. A total of thirty studies were excluded due to lack of inclusion criteria, missing data and multiple publications. All included trials were published after 2004, and vinorelbine plus cisplatin (NP) was the most common chemotherapy regimen (19/29,65.5%), and the remainder included paclitaxel plus cisplatin (TP), gemcitabine plus cisplatin (GP), and docetaxel plus cisplatin (DC). Of the 29 trials included in meta-analysis,24 trials were reported as RCTs, and 5 trials didn't describe clearly the methods of grouping. Of the 24 trials claimed to be RCTs, the randomization procedure was described clearly and was true in only 5 trials(random digital table was adopted), 15 trials stated that subjects were "randomized" without describing the randomization method or procedures, 4 trials stated that methods that were not truly randomized were used. According to the modified Jadad scale, 10 studies were of high quality, with a quality score of 4 or above 4, and the rest were of low quality, with a quality score of 3 or under 3. Characteristics and quality of all included studies are presented in table 2.

**Table 2:** Study characteristics and quality

Studies (Author)	Year	**Chemotherapy regimen**^**a**^		Number (T/C)	Type of Assessable Outcomes	**Jadad Scores**^**b**^
					
		treatment	control			
Hao XL[20]	2008	NP+SFI	NP	60/68	WBC/HB/PLT/nausea and vomiting toxicity, KPS	3
Wang K[21]	2007	NP+SFI	NP	18/18	tumor response, WBC/PLT toxicity, KPS	3
Kang GY[22]	2006	NP+SFI	NP	36/36	tumor response, WBC/PLT/HB/nausea and vomiting toxicity, KPS	2
Gong ZM[23]	2008	NP+SFI	NP	33/32	tumor response, WBC/PLT/HB/nausea and vomiting toxicity, KPS	2
Wang XY[24]	2007	NP+SFI	NP	35/34	tumor response, WBC/PLT/HB/nausea and vomiting toxicity,	4
Wang YZ[25]	2007	NP+SFI	NP	28/27	tumor response, KPS, WBC/PLT/HB toxicity	3
Li TW[26]	2009	NP+SFI	NP	36/33	tumor response, the KPS	4
Li Y[27]	2007	NP+SFI	NP	44/43	tumor response, WBC/PLT/nausea and vomiting toxicity,	4
Lv J[28]	2008	NP+SFI	NP	40/40	tumor response, WBC/PLT/HB/nausea and vomiting toxicity, KPS	4
Zhao ZY[29]	2007	NP+SFI	NP	35/34	tumor response, WBC/PLT/HB nausea and vomiting toxicity,	4
Geng L[30]	2004	NP+SFI	NP	25/15	tumor response, KPS	2
Yu QZ[31]	2007	NP+SFI	NP	30/32	tumor response, KPS	4
Liu CL[32]	2004	NP+SFI	NP	60/60	tumor response, WBC/PLT/HB toxicity	2
Liu PH[33]	2007	NP+SFI	NP	30/30	tumor response, KPS	1
Pan YK[34]	2008	NP+SFI	NP	45/45	tumor response, WBC/PLT/HB toxicity	2
Zheng JH[35]	2009	NP+SFI	NP	42/42	tumor response, WBC/PLT/HB/nausea and vomiting toxicity	4
Miao SR[36]	2010	NP+SFI	NP	38/41	tumor response, the KPS, WBC/PLT/nausea and vomiting toxicity	3
Li YQ[37]	2010	NP+SFI	NP	43/42	KPS	5
Geng D[38]	2007	NP+SFI	NP	42/26	tumor response, WBC/the nausea and vomiting toxicity	2
Zou Y[39]	2005	TP+SFI	TP	24/24	tumor response, KPS	3
Luo SZ[40]	2006	TP+SFI	TP	25/25	tumor response, KPS, WBC/PLT/nausea and vomiting toxicity	2
Luo SW[41]	2007	TP+SFI	TP	30/30	tumor response, WBC/PLT/HB/nausea and vomiting toxicity, KPS	2
Zhang FL[42]	2008	TP+SFI	TP	30/30	tumor response, WBC/PLT/HB/nausea and vomiting toxicity, KPS	3
Zhao YX[43]	2009	TP+SFI	TP	40/40	tumor response, KPS	2
Yu F[44]	2007	DC+SFI	DC	30/30	tumor response, WBC/PLT/HB toxicity	4
He WJ[45]	2008	GP+SFI	GP	35/35	tumor response, WBC/PLT/HB/nausea and vomiting toxicity, KPS	3
Liang K[46]	2010	GP+SFI	GP	39/37	tumor response, KPS,	2
Chen J[47]	2007	TP/NP+SFI	TP/NP	41/39	tumor response, KPS	2
Wu L[48]	2004	TP/NP+SFI	TP/NP	30/30	tumor response, WBC toxicity	5

Abbreviations: SFI, shenqi fuzheng injection; NP, vinorelbine, cisplatin; TP, paclitaxel, carboplatin; DC, docetaxel cisplatin; GP, gemcitabine cisplatin;T/C, treatment group/control group; KPS, Karnofsky Performance status; WBC, white blood cell; PLT, platelet; HB, hemoglobin;

a: all patients included in studies in both groups received systemic chemotherapy therapy, and no patients received surgery and radiation, The only difference between the two groups was whether they received SFI.

**b: modified Jadad scale was used**.

### The result of meta-analysis for Objective tumor response

**Figure 1:**
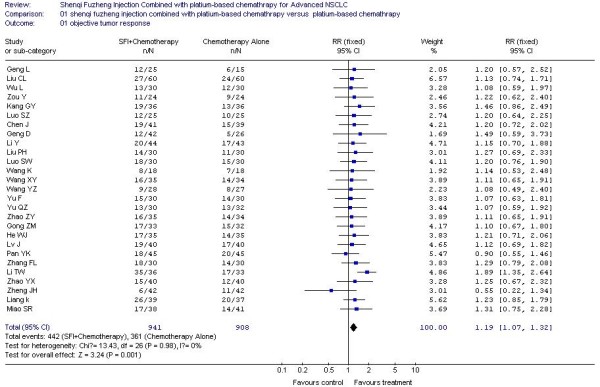
**Forest-plot of objective tumor response**.

In the 29 included trials, the objective tumor response was reported by 27 trials [21-36,38-48], which included 1,849 patients. Meta-analysis showed there was a statistically significant higher tumor response rate (RR, 1.19; 95% CI, 1.07 to 1.32; P = 0.001; Figure 1) in the SFI combined with platinum-based chemotherapy treatment group compared with the platinum-based chemotherapy control group, which meant the significant 19% increase in the RR for the response rate was attributable to the SFI combined with platinum-based chemotherapy treatment group. Because heterogeneity may not lie in the different studies(P = 0.98) in this meta-analysis, the fixed-effect model was used.

### The result of meta-analysis for Performance status

**Figure 2:**
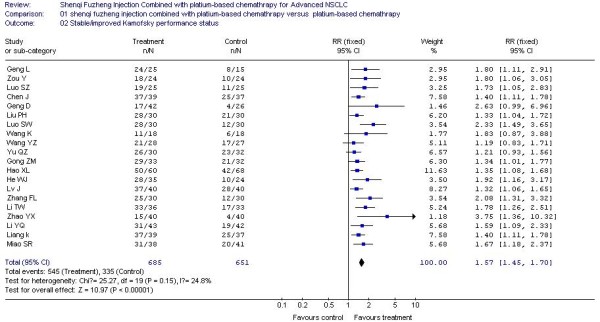
**Forest-plot of stabled/improved Kamofsky performance status**.

The rates of improved or stable performance status were reported in 20 trials [20,21,23,25,26,28,30,31,33,36-43,45-47], which included 1336 patients. Meta-analysis showed there was a statistically significant higher rate of improved or stable performance status (RR, 1.57; 95% CI, 1.45 to 1.70; P < 0.00001; Figure 2) when the SFI combined with platinum-based chemotherapy treatment group was compared with the platinum-based chemotherapy control group, which meant the significant 57% increase in the RR for the rate of improved or stable performance status was attributable to the SFI combined with platinum-based chemotherapy treatment group. For the same reason as objective tumor response, the fixed-effect model was performed in this meta-analysis.

### The result of meta-analysis for grade 3 or 4 WBC, PLT, HB, Nausea and Vomiting Toxicity

**Figure 3:**
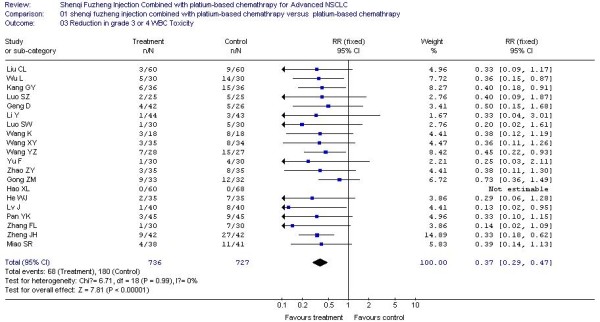
**Forest-plot of grade 3 or 4 WBC toxicity**.

**Figure 4:**
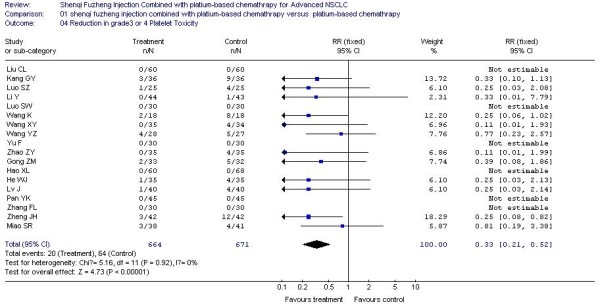
**Forest-plot of grade 3 or 4 PLT toxicity**.

**Figure 5:**
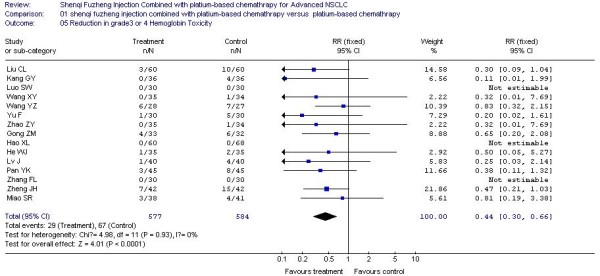
**Forest-plot of grade 3 or 4 HB toxicity**.

**Figure 6:**
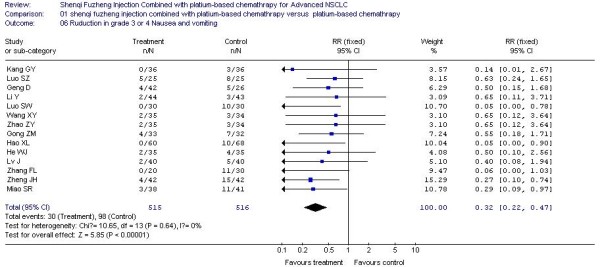
**Forest-plot of grade 3 or 4 nausea and vomiting toxicity**.

In all included studies, 20 trials [20-25,27-29,32,34-36,38,40-42,44,45,48] reported the number of patients with grade 3 or 4 white blood cell (WBC) toxicity, 18 trials [20-25,27-29,32,34-36,40-42,44,45] reported the number of patients with grade 3 or 4 platelet (PLT) toxicity, 15 trials [20,22-25,28,29,32,34-36,41,42,44,45] reported the number of patients with grade 3 or 4 hemoglobin (HB) toxicity and 14 trials [20,22-24,27-29,35,36,38,40-42,45] reported the number of patients with grade 3 or 4 nausea and vomiting. The rate of severe chemotherapy toxicity was calculated for WBC, PLT, HB, nausea and vomiting, and then meta-analyses were performed. As shown in Figures, the results indicated there was statistically significant lower severe toxicity for WBC (RR, 0.37; 95% CI, 0.29 to 0.47; P < 0.00001; Figure 3), PLT (RR, 0.33; 95% CI, 0.21 to 0.52; P < 0.00001; Figure 4), HB (RR, 0.44; 95% CI, 0.30 to 0.66; P < 0.0001; Figure 5) and nausea and vomiting (RR, 0.32; 95% CI, 0.22 to 0.47; P < 0.00001; Figure 6) when the SFI plus platinum-based chemotherapy treatment group was compared with the platinum-based chemotherapy control group.

### The result of sensitivity analysis

The sensitivity analysis revealed that with low quality studies excluded, the summary RR and 95% CIs for above outcomes were still similar to the results before they were excluded (table 3), which indicates that the results of our study are reliable and believable.

**Table 3:** Sensitivity analysis of this study

Outcomes		All Studies			Good Quality Studies			
		
	N	Patients	RR (95%CI)	P	N	Patients	RR (95%CI)	P
Tumor response	27	1849	1.19[1.07,1.32]	0.001	9	640	1.16[0.98,1.38]	0.08
KPS	20	1336	1.57[1.45,1.70]	<0.00001	4	296	1.45[1.25,1.68]	<0.00001
WBC	20	1463	0.37[0.29,0.47]	<0.00001	7	510	0.32[0.21,0.48]	<0.00001
PLT	18	1335	0.33[0.21,0.52]	<0.00001	6	450	0.21[0.09,0.50]	0.0005
HB	15	1161	0.44[0.30,0.66]	<0.001	5	362	0.37[0.19,0.72]	0.003
Nausea and Vomiting	14	1031	0.32[0.22,0.47]	<0.00001	5	389	0.41[0.22,0.77]	0.006

Abbreviations: KPS, Karnofsky Performance status; WBC, white blood cell; PLT, platelet; HB, hemoglobin; N, the number of trials.

### The result of publication bias analysis

**Figure 7:**
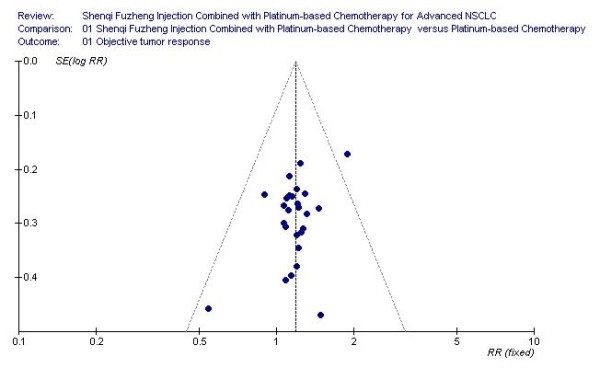
**Funnel plot, based on studies with data on objective tumor response**.

Figure 7 is the funnel plot based on studies with data on objective tumor response, which is asymmetrical, and indicates that publication bias may have existed in our study. The result of Egger's test also suggested an evidence of publication bias (*P *= 0.016).

## Discussion

In medicine, systematic reviews and meta-analysis form the core of a movement to ensure that medical treatments are based on the best available empirical data. One important advantage for meta-analysis is that it can enable the user to perform statistical synthesis and then it can be used to enhance the statistical power to obtain a more accurate conclusion [49]. Thus, to systematically evaluate whether SFI increases the efficacy and decreases the toxicity when combined with platinum-based chemotherapy for advanced NSCLC, the authors conducted a systematic review. The results suggested that SFI intervention may enhance tumor response, improve performance status, and reduce chemotherapy toxicity, when compared with platinum-based chemotherapy alone. This is the first systematic review of SFI for advanced NSCLC and the results can provide important references about how to reduce toxicity and enhance the curative effect of platinum-based chemotherapy. In China, it is common to use SFI to treat advanced NSCLC, but no relevant articles or evaluations have been published in the English medical journals, hence reducing its worldwide validity. This study may prove useful for supplementing the evidence for the use of SFI in the treatment of advanced NSCLC.

Shenqi Fuzheng Injection is concocted from Radix Astragali(huangqi) and Radix Codonopsis(dangshen). These two kind of Chinese medicinal herbs have been used in China and some other Asia countries as herbal medicines for many years. Of them, Radix Astragali is usually used as an immunomodulating agent in the treatment of immunodeficiency diseases and to alleviate the adverse effects of chemotherapeutic drugs [50,51]. Radix Codonopsis is usually used to treat dyspepsia, fatigue, bronchitis, cough, inflammation and so on, and its pharmacological activities such as antifatigue and immunomodulatory activities were also reported[52]. SFI is developed from Radix Astragali and Codonopsis, which suggests that its effect in the treatment of NSCLC may be related with the above pharmacological activities of Radix Astragali and Codonopsis. However, what are the specific immunological and cytotoxic mechanisms? what are main effective components? Do the interactions between medicines or components exist? These questions are not clear and require further investigation.

This systematic review also has limitations. First, allocation concealment and blinding were not described in all included trials, which may result in the emergence of bias, and the overestimation of the efficacy of the treatment group. Second, much of the data on the patients' survival was not reported in the included studies, thus the influence that SFI combined with platinum-based chemotherapy had on survival could not be analyzed by this systematic review. Third, funnel plot and Egger's test suggested publication bias may exist. Given above reasons, the evidence from this study may be insufficient, and should be carefully disseminated to the medical community. However, we all know it is difficult and expensive to carry out clinical trials on advanced NSCLC patients and large, placebo-controlled, double-blind studies are almost impossible. Therefore, trials with above questions may exist in many countries and may be permitted to some extent, but still provide helpful information for clinical practice and drug development. Now it has been increasingly recognized that Western medicine may not be the answer for the treatment of all diseases and sometimes alternative medicines or treatment regimes may prove successful. Therefore, though SFI is a kind of traditional Chinese medicine, the results of this systematic review suggested it may play an important role in the treatment of advanced NSCLC.

## Conclusions

In conclusion, in this systematic review evidence was found that SFI intervention may increase the efficacy and reduce the toxicity when combined with platinum-based chemotherapy for advanced NSCLC, which would provide important references about how to reduce toxicity and enhance the curative effect of platinum-based chemotherapy for advanced NSCLC. However, limitations remain and the results needs to be further verified by more high-quality trials.

## Competing interests

The authors declare that they have no competing interests.

## Authors' contributions

JD, ZZ conceived the study, JD, SYS, MYW, ZZ participated in protocol design. JD, SYS ran the searches and abstracted data. JD performed the analysis. JD, SYS, MYW, ZZ wrote and approved the manuscript.
